# Flap Reconstruction Outcome Following Surgical Resection of Soft Tissue and Bone Sarcoma in the Setting of (Neo)adjuvant Therapy: A Sarcoma Center Experience

**DOI:** 10.3390/cancers15092423

**Published:** 2023-04-23

**Authors:** Ioana Lese, Crinu Baesu, Isabel Arenas Hoyos, Michael-Alexander Pais, Frank Klenke, Attila Kollar, Codruta Ionescu, Mihai Constantinescu, Radu Olariu

**Affiliations:** 1 Department of Plastic and Hand Surgery, Inselspital, Bern University Hospital, 3008 Bern, Switzerland; 2 Department of Orthopedic Surgery, Inselspital, Bern University Hospital, 3010 Bern, Switzerland; 3 Department of Medical Oncology, Inselspital, Bern University Hospital, 3012 Bern, Switzerland; 4 Department of Radiation Oncology, Inselspital, Bern University Hospital, 3012 Bern, Switzerland

**Keywords:** sarcoma, soft tissue, bone, reconstructive surgery, free and pedicled flap, neoadjuvant and adjuvant therapy

## Abstract

**Simple Summary:**

Soft tissue and bone sarcomas are a diverse group of aggressive tumors. Lately, the shift in their management, with an emphasis on limb salvage, has deemed the involvement of reconstructive surgeons an integral part of their multidisciplinary treatment. We present our experience with 90 free and pedicled free tissue transfers in the reconstruction of sarcomas at a major sarcoma center over a 5-year period: diabetes, alcohol consumption and male gender were associated with increased wound healing problems, preoperative chemotherapy significantly increased the occurrence of early infection, while preoperative radiotherapy was associated with a higher incidence of lymphedema. Reconstructive surgery with either pedicled or free tissue transfer after sarcoma resection is reliable, but a higher complication rate is to be expected with neoadjuvant therapy and with certain comorbidities.

**Abstract:**

Background: Soft tissue and bone sarcomas are heterogeneous groups of malignant tumors. The shift in their management, with an emphasis on limb salvage, has deemed the involvement of reconstructive surgeons an integral part of their multidisciplinary treatment. We present our experience with free and pedicled flaps in the reconstruction of sarcomas at a tertiary referral university hospital and major sarcoma center. Materials and Methods: All patients undergoing flap reconstruction after sarcoma resection over a 5-year period have been included in the study. Patient-related data and postoperative complications were collected retrospectively, ensuring a minimum follow-up of 3 years. Results: A total of 90 patients underwent treatment with 26 free flaps and 64 pedicled flaps. Postoperative complications occurred in 37.7% of patients, and the flap failure rate was 4.4%. Diabetes, alcohol consumption and male gender were associated with increased early necrosis of the flap. Preoperative chemotherapy significantly increased the occurrence of early infection and late dehiscence, while preoperative radiotherapy was associated with a higher incidence of lymphedema. Intraoperative radiotherapy was associated with late seromas and lymphedema. Conclusions: Reconstructive surgery with either pedicled or free flaps is reliable, but it can be demanding in the setting of sarcoma surgery. A higher complication rate is to be expected with neoadjuvant therapy and with certain comorbidities.

## 1. Introduction

Soft tissue and bone sarcomas are a heterogeneous group of malignant tumors originating from mesenchymal cells. The European incidence for soft tissue sarcomas is evaluated at 4.7 per 100,000 per year, with 50–60% occurring in the extremities, while bone sarcomas account for 0.8 per 100,000 per year [1,2]. Modern treatment strategies in extremity sarcomas emphasize limb preservation since available data showed no difference in overall or disease-free survival when comparing patients undergoing limb salvage or amputation [3,4]. Therefore, the involvement of plastic and reconstructive surgeons has become an integral part of the multidisciplinary treatment of these complex cases, with reconstruction being required for many extremity sarcomas treated with limb preservation surgery [5,6,7,8,9,10]. The need for soft tissue reconstruction after primary resection of soft tissue sarcomas has been reported in the literature up to 33–47% of the patients, while the need for soft tissue reconstruction after previously unplanned resections of soft tissue sarcomas vary between 47–89% of the patients in specialized sarcoma centers [11,12,13]. When loco-regional flaps are deemed insufficient, free microsurgical transfers are needed to ensure a limb-preserving approach.

As previously reported by Slump et al., the use of flaps increases the complexity of the procedure but does not increase postoperative complications [14]. Moreover, no differences were reported when comparing complications between patients undergoing reconstruction with pedicled or free flaps [15]. Nevertheless, there are multiple variables that might influence the outcome of the reconstruction, such as (neo)adjuvant therapy, age, body mass index (BMI) and other comorbidities [16,17,18,19]. Especially in the cases where neoadjuvant therapy is employed, free tissue transfer is preferred since nonirradiated tissue is brought into the defect, and usually, the anastomoses are performed outside the irradiation field.

In this study, we present our experience with free and pedicled flaps in the reconstruction of soft tissue and bone sarcomas over a 5-year period at a tertiary referral university hospital and sarcoma center.

## 2. Materials and Methods

All patients undergoing flap reconstruction after sarcoma resection between January 2014 and December 2018 in the Department of Plastic and Reconstructive Surgery, University Hospital in Bern, Switzerland, were included in this study in order to ensure a minimum follow-up of 3 years. The following patient-related data were retrospectively collected for each patient: age at the time of surgery, gender, American Society of Anaesthesiologists (ASA) classification, alcohol consumption, smoking status, body mass index (BMI), medical comorbidities (high blood pressure, diabetes mellitus, heart failure, coronary artery disease, cardiac arrhythmias and others), tumor characteristics (soft tissue/bone sarcoma, location, maximal diameter), surgical details (type of referral—primary/recurrence, type of flap), the use of neoadjuvant and adjuvant therapy (radiotherapy and/or chemotherapy), intraoperative radiotherapy (IORT) and disease outcome (local recurrence and distant metastasis). The choice of the pedicled or free flap was individualized. Usually, patients with neoadjuvant radiotherapy and extensive tumors underwent reconstruction with a free flap and the anastomoses were performed, when possible, outside of the radiation field.

All postoperative complications, especially regarding flap failures, were recorded. The surgical site complications (infection, partial flap necrosis, hematoma, seroma, dehiscence and others) were divided into early and late complications, depending on whether they ensued before or after 30 days postoperatively. Complications appearing early and lasting past the 30th postoperative day were still considered early complications. Moreover, major complications were defined as events requiring revision surgery, while minor complications were treated conservatively. Differences in patients with and without postoperative complications were assessed with the student’s *t*-test or the Mann-Whitney U test for the continuous variables, depending on data distribution, while Pearson’s χ2 test and Fischer’s exact test were conducted for the categorical variables. Statistical analysis was performed using SPSS 23.0 (SPSS Inc., Chicago, IL, USA).

## 3. Results

### 3.1. Patient and Tumor Characteristics

Out of the 202 patients undergoing sarcoma resection over the course of 5 years, 90 patients required reconstruction with either a pedicled or free flap, excluding patients with atypical lipomatous and cartilaginous tumors. These tumors, although strictly speaking, are malignant, behave more like benign tumors and, therefore, seldom require radiation and reconstructive surgery. Patients and tumor characteristics are presented in Table 1 and Table 2. The median age of the included patients was 58.5 years, ranging from 11 to 87 years old. Most of the patients (85.6%) presented with a primary tumor, while 13 patients were referred with recurrent sarcoma. Tumor maximal diameter had a median of 8.35 cm (range 1–26 cm). Eighty-five patients (94.4%) had at least one comorbidity, with arterial hypertension being the most common comorbidity (44.4% of the patients), followed by coronary heart disease (10% of the patients). Alcohol consumption was reported by 24.4% of the patients, while active smoking was recorded by 31.1% of the patients. There were 78 patients with soft tissue sarcomas, while the most common location of the sarcomas was the lower extremity (42.2%), followed by the trunk (30%). Among the soft tissue sarcomas, seven skin sarcomas were recorded (5 cases of dermatofibrosarcoma protuberans and 2 cases of pleomorphic dermal sarcomas). Out of the 12 bone sarcomas, 8 were osteosarcomas, 1 was Ewing sarcoma, while the rest were represented by chondrosarcomas. Neoadjuvant therapy was recorded in 39 patients (43.3%), with neoadjuvant radiotherapy being the most frequent one (25 patients—27.8%). The median time between neoadjuvant radiotherapy and surgery was 32 days (range of 17–48 days). Intraoperative radiotherapy was applied in 54 patients (60%), with a standard dose of 10 Grays. Adjuvant therapy was reported in 43 patients (47.8%).

There were 26 free flaps and 64 pedicled/loco-regional flaps, as outlined in Table 3. The most common pedicled flaps were the vertical rectus abdominis muscle (VRAM) and medial gastrocnemius flap, while the most common free flaps were the antero-lateral thigh (ALT), latissimus dorsi myocutaneous and gracilis muscle flap. All bone flaps were free flaps, represented by three fibula-free flaps and one calcaneus-free flap. Two free flaps recorded vascular compromise with postoperative thrombosis, out of which one was saved (lateral arm flap). The lost gracilis muscle flap was replaced by another free gracilis muscle flap, with subsequent uncomplicated healing. There were three flap failures in the pedicled flap group: a VRAM flap for the thigh, a supraclavicular flap for the head and neck region, and a propeller flap for the lower extremity. While the defect left by the debridement of the first flap was managed through local muscle flaps, the second one needed a free ALT flap for definitive wound closure. The defect left after the removal of the propeller flap was closed in the end with a skin graft. Overall, with one free flap and three pedicled flaps lost, the flap failure rate was 4.44%. There was no statistically significant difference in tumor size between the patients undergoing reconstruction with a pedicled or free flap.

### 3.2. Postoperative Complications

Thirty-four patients (37.7%) recorded postoperative complications. Early complications ensued in 18 patients (20%), while late complications were recorded in 26 patients (28.9%), with ten patients having both early and late complications. Early complications of the donor side were represented by necrosis, bleeding and seroma, with the latter one being the most cumbersome. The 2 cases of seroma ensued at the harvesting site of latissimus dorsi myocutaneous flaps, and in both cases, a surgical revision was required. The most common early recipient site complication was partial flap necrosis, ensuing in 6 patients, out of which four had to undergo revision surgery. Infection was the second most common early recipient site complication, being recorded in 6 patients, with 5 of them requiring revision surgery. Late donor site complications included infection, seroma and lymphedema, with the former being the most frequent. Surprisingly, late complications of the recipient site surpassed the number of early complications. Wound dehiscence was the most common one, with 6 out of the 11 patients requiring revision surgery. Lymphedema was reported as a minor complication in 10 patients, while infection developed in 9 patients, with 6 of them recording a major complication. A more detailed description of the early and late complications of the donor and recipient site is presented in Table 4. Table 5 lists the complication rates based on the type of flap that was used. However, we did not find any significant differences in whether a free flap or a pedicled flap was used. Moreover, sarcoma location also did not seem to influence the complication rates.

The presence of diabetes, alcohol consumption and male patients were associated with increased early partial necrosis of the flap at the recipient site (28.6% vs. 4%, *p* = 0.011; 20% vs. 4.5%, *p* = 0.046 and 14.9% vs. 0%, *p* = 0.013, respectively), while flap failure was significantly more common with alcohol consumption (20% vs. 0%, *p* = 0.002). A BMI ≥ 30 kg/m^2^ was significantly associated with a higher overall complication rate: 70.6% compared to 43.1% in patients with a BMI < 30 kg/m^2^ (*p* = 0.041).

When looking at tumor characteristics, bone sarcomas were associated with a higher rate of late complications: 58.3% compared to 24.4% in soft tissue sarcomas (*p* = 0.034). Neoadjuvant therapy also appeared to increase the complication rate: preoperative chemotherapy significantly increased the occurrence of early infection and late dehiscence at the recipient site (21.4% vs. 4%, *p* = 0.047 and 35.7% vs. 6.7%, *p* = 0.008, respectively), while preoperative radiotherapy was associated with a higher incidence of lymphedema of the recipient site (23.1% vs. 6.3%, *p* = 0.023). Patients with intraoperative radiotherapy recorded late seromas in 11.3% of the cases, while the other patients did not have this complication (*p* = 0.037). Moreover, lymphedema of the recipient site was also more frequent in patients receiving intraoperative radiotherapy (16.7% vs. 2.8%, *p* = 0.04). Patients with early complications (up to 30 days postoperatively) had a significant delay until the start of postoperative radiotherapy: 88 days (range 41–147 days) vs. 53 days (range 10–83 days), *p* = 0.017. A more detailed description of the complications, based on the chemotherapy regimen is presented in Table 6. Table 7 depicts a more detailed description of the complications at the recipient site based on radiotherapy regimen (adjuvant radiotherapy refer mostly to adjuvant radiotherapy, either immediate, such as IORT, or late standard postoperative radiotherapy, including therefore IORT alone, IORT with postoperative radiotherapy and postoperative radiotherapy alone). The one case with preoperative, IORT and postoperative radiotherapy is not included in the table, but this patient did not have any postoperative complications.

### 3.3. Disease Outcome

A local recurrence was detected in 11 patients (12.2%), with only one of the patients having a pathologically confirmed R1 resection during the initial operation, due to a skip lesion. There were no statistically significant differences in the minimal clear margins between patients with and without a local recurrence (3 mm median value/64 mm mean, 0–17 mm range, and 4 mm medial value/63 mm mean, 1–40 mm range, respectively). However, 10 of the patients had a high-grade tumor. Distant recurrence was diagnosed in 32 patients (35.6%), out of which 23 were locally disease free. 6 patients underwent palliative care, while the rest had multimodality treatment with radiotherapy, chemotherapy, surgery, or a combination of them. At the 3-year follow-up, 19 patients (21.2%) died due to the progression of the disease, while 4 patients died due to other reasons. 14 patients (15.6%) were alive with disease, while the rest of the patients (53 patients—58.9%) were alive without disease.

## 4. Discussion

Soft tissue and bone sarcomas are rare malignant tumors that arise from mesenchymal cells, most commonly affecting the extremities. Amputation was previously thought to be the best treatment when considering local recurrence and survival rates in this localization. However, improvements in (neo)adjuvant radiotherapy, imaging and surgical technique, together with an enhanced emphasis on multidisciplinary approach have paved the path to improvements in the treatment of sarcomas. Preoperative, neoadjuvant radiotherapy, potentially combined with IORT, is currently preferred over postoperative radiotherapy due to the lower dose of radiation and the reduction of the radiation volume [20]. Moreover, reconstructive surgery could allow a higher radiation dosage when it is known that afterwards, healthy, nonirradiated tissue will be employed for coverage. However, an individualized decision should pe taken in every sarcoma patient when discussing pre- versus postoperative radiotherapy. Nonetheless, in the setting of reconstructive therapy when flaps are required after sarcoma resection, preoperative radiotherapy is known to be associated with a higher rate of wound complications that could delay recovery and perhaps compromise further recommended treatment [7,16,17,18,21,22,23,24]. Interestingly, our study did not identify an association between preoperative radiotherapy and early wound complications, as would be expected based on current literature. However, we recorded a higher rate of complications arising after the 30th postoperative day, defined as late complications. Specifically, lymphedema had a higher incidence in our cohort. It is important to mention that all patients with preoperative radiotherapy also underwent IORT. Since lymphedema is usually reported after postoperative radiotherapy, pertaining to the long-term group of complications, we looked closer at the 27 patients that underwent preoperative radiotherapy. One of these patients received also postoperative radiotherapy of a new detected skip lesion outside of the initial irradiation volume, therefore he was excluded as a confounder when looking at the incidence of lymphedema. Lymphedema is a major concern and can impact significantly the quality of life of sarcoma patients due to the prolonged therapy and associated frequent infections [25]. However, data on the effect of neoadjuvant radiotherapy and IORT and the incidence of lymphedema are scarce and unclear [26]. Some recent attempts at using lymphatic surgery techniques to treat the effects of lymphatic drainage disruption by sarcoma surgery have shown promising results: soft tissue transfers with lymphatic tissue preservation, as well as lymphatic flow-through flaps were employed [27]. Since our data suggests that patients receiving preoperative therapy have an increased risk of lymphedema, it may be worthwhile considering surgical lymphatic restoration techniques when reconstructing these defects in order to prevent its appearance in the postoperative period.

Moreover, in our study, patients receiving IORT had a higher rate of late seroma at the recipient site. While this complication hasn’t been frequently reported in the literature, various authors point out to the possibility of increased late toxicity with IORT, despite the high limb preservation rates with good functional results [21,28]. Additionally, with the advent of protocol-based pre and postoperative chemotherapy, the cure rate of osteosarcomas and Ewing sarcomas is estimated to be over 60%, which is a clear improvement when compared to the <20% value reported in the pre-chemotherapy era [1]. Nonetheless, this type of neoadjuvant therapy is also associated with wound complications, such as wound dehiscence and infections [29], paralleling the results of this study in which early infection and late dehiscence at the recipient site were shown to be higher in patients receiving preoperative chemotherapy.

When looking at comorbidities, diabetes is a well-known risk factor for wound healing problems in the majority of free microsurgical procedures [30,31,32], as it was also reported by other authors when looking specifically at sarcoma surgery [16,33,34]. We have also observed a higher rate of early necrosis at the recipient site in these patients when compared to patients without a history of diabetes: 28.6% vs. 4%. Moreover, in our study, obesity, defined as a BMI ≥ 30 kg/m^2^, was also associated with a higher overall complication rate, in holding with the results of other literature reports [14]. While in our series we did not see an increased complication rate in patients with a history of smoking, we detected a statistically significant higher flap failure rate in patients with active alcohol consumption, which has not been reported yet in sarcoma surgery.

Free tissue transfer is often seen as a complex procedure linked to frequent complications due to the need to perform microvascular anastomosis, which would also increase the patients’ anesthesia time. While it is true that it adds to the difficulty of the surgical procedure itself, we also have to acknowledge the fact that pedicled, loco-regional flaps might negatively influence the functional outcome of the reconstructed region, since the tissue surrounding the sarcoma resection undergoes extensive dissection. Additionally, the pedicles of loco-regional flaps may be located in areas subjected to radiotherapy and/or surgical manipulation during resection, adding to potential risks of flap compromise [24]. Our study did not identify any differences between free and pedicled flaps when looking at complications and flap failures, similar to the results reported by other authors, considering them a safe and reliable reconstructive method. Slump et al. recorded postoperative complications in 32% of the patients undergoing sarcoma resection and reconstruction with pedicled flaps, while 38% of the patients undergoing reconstruction with free tissue transfer displayed postoperative complications [15]. Reconstructive surgery after sarcoma resection aims at maximizing functional outcome. However, a thorough preoperative counseling of the patients is necessary in order for them to understand not only the risks, but also the benefits of the recommended reconstructive techniques.

Local recurrence rates seem to correlate with the grade of the sarcoma, as recent literature shows [35,36]. The 12.2% local recurrence rate recorded in our series seems to be in line with those results, since 10 out the 11 patients with local recurrence had a high-grade sarcoma. While there are studies in the literature describing recurrence rates as high as 39.8%, there are also studies reporting rates as low as 5%. However, the median maximal diameter of the resected sarcomas (8.35 cm) in our patients is among the higher end of the tumor sizes, so this might be an explanation for our results [35,36,37].

The limitations of our study consist in the limited number of patients and the retrospective design of the study. However, the relatively small number of patients is not unexpected since bone and soft tissue sarcoma are a rare and heterogenous group that are treated only in highly specialized centers like ours. Therefore, an underpowered multivariate logistic regression analysis was purposely forgone.

## 5. Conclusions

While reconstructive surgery with either pedicled or free flaps can be demanding in the setting of sarcoma surgery, our study shows good results compared to the available literature. A higher complication rate is to be expected with neoadjuvant therapy and a few well known comorbidities. Interestingly, our results draw attention to the association between preoperative/intraoperative radiotherapy and late complications, such as lymphedema and seroma. These results could be helpful for pre-operative patient counselling and the provision of accurate risk assessment when discussing reconstructive techniques in sarcoma surgery.

## Figures and Tables

**Table 1:** Patients’ characteristics.

Characteristic		N (%)
Gender	Female	42 (46.7)
	Male	48 (53.3)
BMI	<30 kg/m^2^	70 (77.8)
	≥30 kg/m^2^	20 (22.2)
ASA	1	4 (4.4)
	2	38 (42.2)
	3	43 (47.8)
	4	5 (5.6)
Alcohol consumption	No	68 (75.6)
	Yes	22 (24.4)
Smoking	No	62 (68.9)
	Yes	28 (31.1)
Arterial hypertension	No	50 (55.6)
	Yes	40 (44.4)
Diabetes	No	76 (84.4)
	Yes	14 (15.6)
Heart failure	No	85 (94.4)
	Yes	5 (5.6)
Coronary heart disease	No	81 (90)
	Yes	9 (10)
Cardiac Arrhythmias	No	85 (94.4)
	Yes	5 (5.6)
Peripheral arterial occlusive disease	No	89 (98.9)
	Yes	1 (1.1)
Chronic venous insufficiency	No	85 (94.4)
	Yes	5 (5.6)
COPD/Asthma	No	83 (92.2)
	Yes	7 (7.8)

BMI body mass index, COPD chronic obstructive pulmonary disease.

**Table 2:** Tumor characteristics.

Type of sarcoma	Bone	12 (13.3)
	Soft tissue	78 (86.7)
Sarcoma location	Head & Neck	8 (8.9)
	Trunk	27 (30)
	Upper extremity	17 (18.9)
	Lower extremity	38 (42.2)
Neoadjuvant therapy		39 (43.3)
	Chemotherapy	13 (14.4)
	Radiotherapy	26 (28.9)
	Combined radio- and chemotherapy	1 (1.1)
Intraoperative radiotherapy	No	36 (40)
	Yes	54 (60)
Adjuvant therapy		43 (47.8)
	Chemotherapy	17 (18.9)
	Radiotherapy	24 (26.7)
	Combined radio- and chemotherapy	2 (2.2)

**Table 3:** Flap types were performed for reconstruction after soft tissue and bone sarcoma resections.

Flap Type		Pedicled Flaps	Free Flaps
Fasciocutaneous	25	19	6
Muscle	22	17	5
Musculocutaneous	39	28	11
Bone	4	0	4

**Table 4:** Early and late postoperative complications of the donor and recipient site.

Complications	All (%)	Minor (%)	Major (%)
Early complications	18 patients (20)		
Donor site			
Infection	-	-	-
Necrosis	1 (1.1)	1 (1.1)	-
Bleeding/Hematoma	1 (1.1)	1 (1.1)	-
Seroma	2 (2.2)	-	2 (2.2)
Wound dehiscence	-	-	-
Recipient site			
Infection	6 (6.7)	1 (1.1)	5 (5.6)
Partial flap necrosis	7 (7.7)	3 (3.3)	4 (4.4)
Bleeding/Hematoma	4 (4.4)	2 (2.2)	2 (2.2)
Seroma	2 (2.2)	-	2 (2.2)
Wound dehiscence	4 (4.4)	1 (1.1)	3 (3.3)
Late complications	26 patients (28.9)		
Donor site			
Infection	3 (3.3)	2 (2.2)	1 (1.1)
Bleeding/Hematoma	-	-	-
Seroma	2 (2.2)	2 (2.2)	-
Lymphedema	1 (1.1)	1 (1.1)	-
Wound dehiscence	-	-	-
Recipient site			
Infection	9 (10)	3 (3.3)	6 (6.7)
Bleeding/Hematoma	1 (1.1)	-	1 (1.1)
Seroma	6 (6.6)	3 (3.3)	3 (3.3)
Lymphedema	10 (11.1)	10 (11.1)	-
Wound dehiscence	11 (12.3)	5 (5.6)	6 (6.7)

**Table 5:** Complication rates in free and pedicled flaps.

	Free Flaps	Pedicled Flaps
Early complications	6/26 (23.1%)	14/64 (21.9%)
Late complications	6/26 (23.1%)	21/64 (32.8%)
Overall complications	12/26 (46.2%)	28/64 (43.8%)
Flap failure rate	1/26 (3.8%)	3/64 (4.6%)

**Table 6:** Complications at the recipient site based on the chemotherapy regimen.

	No Chemotherapy	Neoadjuvant Chemotherapy	Adjuvant Chemotherapy	Combined Neoadjuvant and Adjuvant Chemotherapy
Early complications	8/68 (11.8%)	2/3 (66.7%)	0/8 (0%)	1/11 (9.1%)
Late complications	14/68 (20.6%)	2/3 (66.7%)	1/8 (12.5%)	3/11 (27.3%)
Overall complications	17/68 (25%)	2/3 (66.7%)	1/8 (12.5%)	4/11 (36.4%)

**Table 7:** Complications at the recipient site based on radiotherapy regimen.

	Neoadjuvant Radiotherapy and IORT	Adjuvant Radiotherapy	No Radiotherapy
**Overall complications**	8/26 (30.8%)	9/31 (29%)	7/31 (22.6%)
**Early complications**	3/26 (11.5%)	4/31 (12.9%)	4/31 (12.9%)
Infection	2/26 (7.7%)	2/31 (6.5%)	2/31 (6.5%)
Necrosis	1/26 (3.8%)	3/31 (9.7%)	3/31 (9.7%)
Bleeding/Hematoma	2/26 (7.7%)	1/31 (3.2%)	1/31 (3.2%)
Seroma	1/26 (3.8%)	1/31 (3.2%)	0/31 (0%)
Wound dehiscence	1/26 (3.8%)	1/31 (3.2%)	2/31 (6.5%)
**Late complications**	7/26 (27%)	8/31 (25.8%)	5/31 (16.1%)
Infection	4/26 (15.4%)	3/31 (9.7%)	2/31 (6.5%)
Bleeding/Hematoma	1/26 (3.8%)	0/31 (0%)	0/31 (0%)
Seroma	3/26 (11.5%)	3/31 (9.7%)	0/31 (0%)
Lymphedema	6/26 (23.1%)	3/31 (9.7%)	1/31 (3.2%)
Wound dehiscence	4/26 (15.4%)	2/31 (6.5%)	4/31 (12.9%)

## Data Availability

The data presented in this study are available on request from the corresponding author. The data are not publicly available due to privacy and ethical reasons.
